# Supplementary material for: Unanchored tri‐NEDD8 inhibits PARP‐1 to protect from oxidative stress‐induced cell death
Source: EMBO J. 2019 Feb 25;38(6):e100024. doi: 10.15252/embj.2018100024 (PMC6418418; doi:10.15252/embj.2018100024)
Supplement: Supplementary file 1 — Expanded View Figures PDF [file EMBJ-38-e100024-s001.pdf]

Expanded View Figures

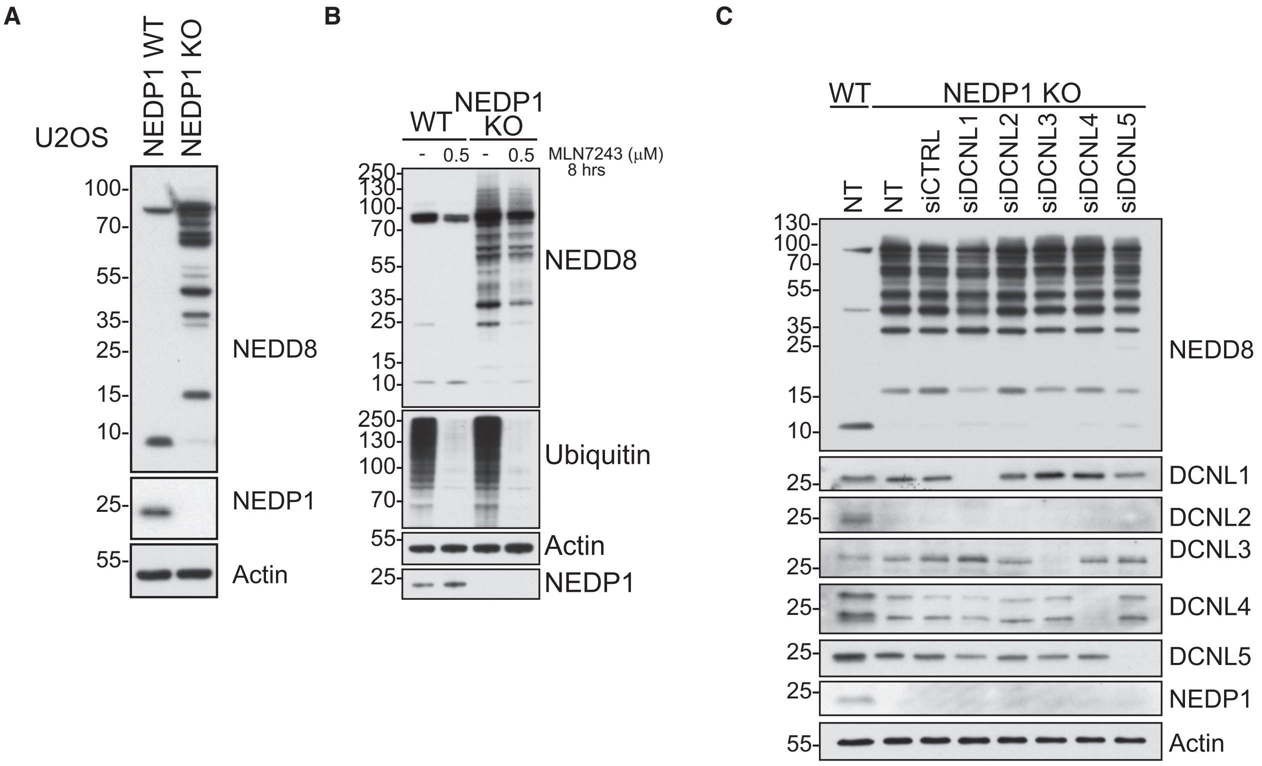

Figure EV1. Generation and analysis of NEDP1 knockout U2OS cells.

A Western blot analysis of whole-cell lysates from U2OS WT and NEDP1 KO cells reveals a loss of free NEDD8 and an accumulation of NEDD8 reactive species in the NEDP1 KO lysate.

B Neddylated species are not dependent on the ubiquitin E1. Western blot analysis of whole-cell lysate from WT and NEDP1 KO HEK 293 cells treated MLN7243 (0.5 μM) for 8 h. The presence of ubiquitylated species is decreased after treatment, but the amount of neddylated species are not significantly reduced.

C Neddylated species are not dependent on the NEDD8 co-E3s DCNL1-5. Western blot analysis of whole-cell lysates from WT and NEDP1 KO HEK 293 cells untreated (NT) or treated with the indicated siRNA for 48 h.

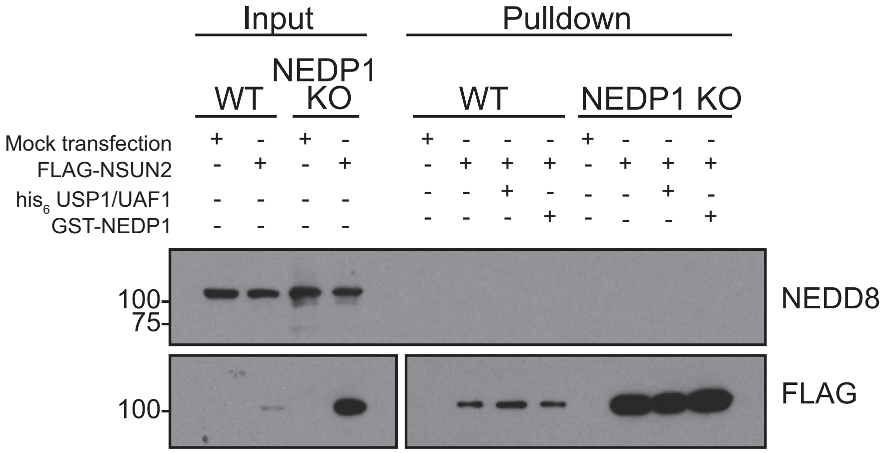

Figure EV2. Neddylation of FLAG-NSUN2 is not readily detected in WT or NEDP1 KO cells.

WT and NEDP1 KO HEK 293 cells were transfected with FLAG-NSUN2 and 48 h later harvested for anti-FLAG immunoprecipitation. Following stringent washes, pulldowns were treated with recombinant GST-NEDP1, the deubiquitinase his_6-USP1/UAF1, or left untreated. After incubation at 30°C for 30 min, reactions were stopped by the addition of LDS sample buffer, and then, samples were processed for Western blot analysis with the indicated antibodies. There is no shift in the anti-FLAG immunoreactive band, which suggests NSUN2 is neither modified by NEDD8 or ubiquitin.

© 2019 The Authors

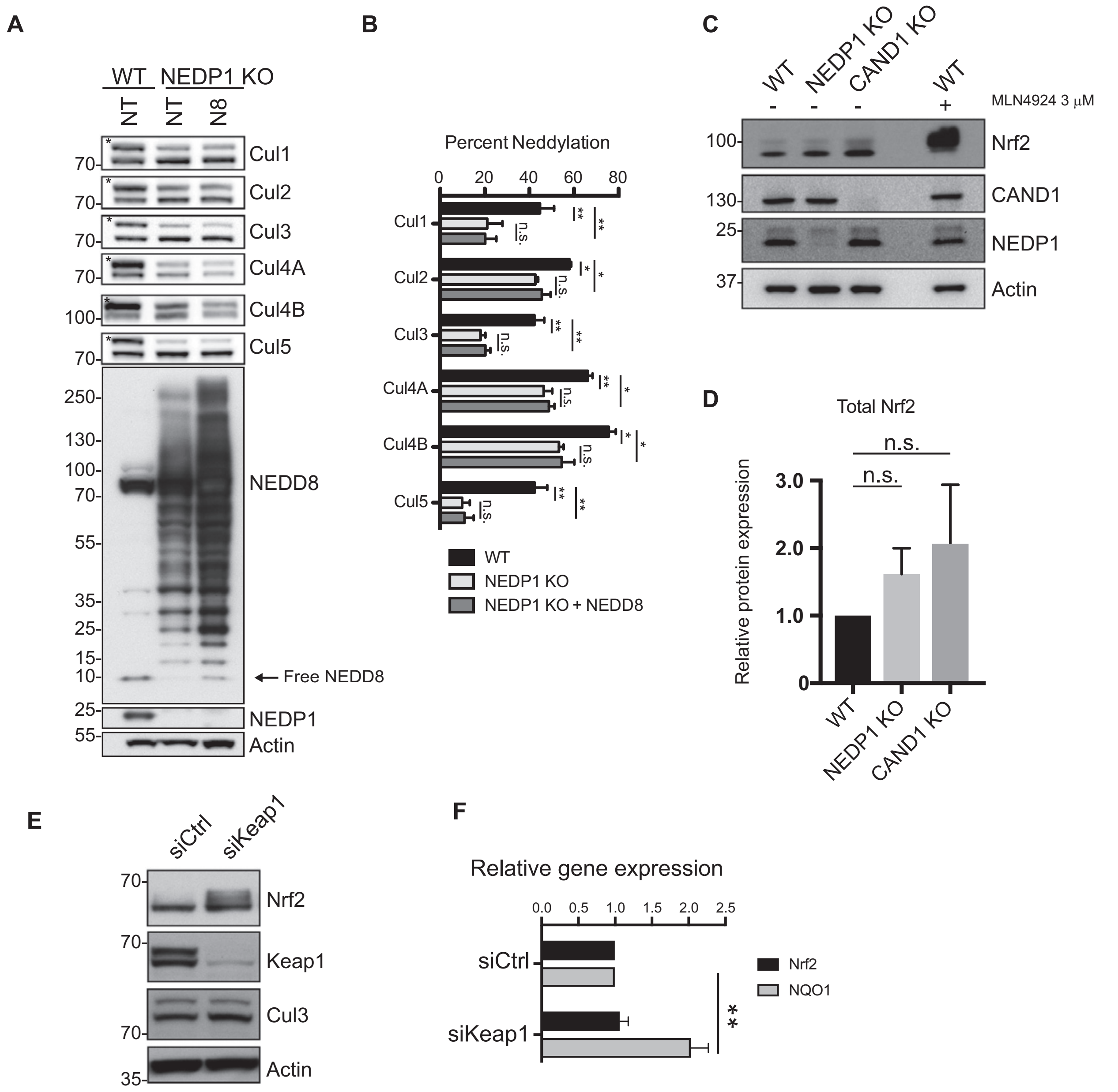

Figure EV3. The cullin neddylation defect in NEDP1 KO cells is not rescued by NEDD8 overexpression.

A Overexpression of NEDD8 is insufficient to rescue the defect in Cullin neddylation in NEDP1 KO cells. Western blot analysis of whole-cell lysates from HEK 293 WT, NEDP1 KO, or NEDP1 KO with transient overexpression of mature NEDD8. The overexpression of NEDD8 results in an increase in the amount of NEDD8 reactive species without a sufficient increase in the level of free NEDD8 to rescue the reduction of Cullin neddylation in NEDP1 KO cells.

B Quantification and graph of the mean ± SEM of the percentage neddylation of each Cullin in (A). One-way ANOVA with Bonferroni *post hoc* test: $n = 3$, $^{*}P < 0.033$, $^{**}P < 0.0021$, n.s. denotes not statistically significant.

C Western blot analysis of the level of Cul3 substrate Nrf2 in HEK 293 WT, NEDP1 KO and CAND1 KO cells. Whole-cell lysates were prepared from the indicated cell lines and analysed by SDS–PAGE and Western blot analysis. In addition, MLN4924 (3 μM) was added to WT cells for 4 h before harvested as a positive control for Nrf2 stabilization. Cul3 substrate Nrf2 is partially stabilized in NEDP1 KO and CAND1 KO cells.

D Quantification and graph of the mean ± SEM of the increase in Nrf2 levels in (C) from NEDP1 KO and CAND1 KO cells. One-way ANOVA with Bonferroni *post hoc* test: $n = 3$, n.s. denotes no statistical significance.

E siRNA-mediated knockdown of Cul3 substrate adaptor Keap1 stabilizes Nrf2 protein levels. U2OS cells were transfected with control siRNA (siCTRL) or siRNA against Keap1 (siKeap1), and 72 h post-transfection, cells were lysed and processed for Western blot analysis.

F Nrf2 stabilization results in the induction of transcription of the Nrf2 response gene NQO1. U2OS cells were transfected with siRNA as in (D), and 48–96 h later, RNA was harvested from cells, reversed transcribed to cDNA and analysed by qPCR for Nrf2 and NQO1 expression. The graph represents mean ± SEM. Unpaired Student's *t*-test: $n = 6$, $^{**}P = 0.0013$.

Figure EV4. PARP-1 activity is reduced in NEDP1 KO cells which is protective against oxidative stress.

A PARP-1 inhibitor DPQ (30 μM) protects WT cells from H_2O_2 treatment. NEDP1 deletion protects U2OS cells from H_2O_2 treatment greater than DPQ, and DPQ does not further protect NEDP1 cells from H_2O_2 treatment. Graphs represent the mean ± SEM of the percent survival compared to untreated cells. Two-way ANOVA with Bonferroni *post hoc* test: $n = 3$, ***$P < 0.0002$.

B FLAG-PARP-1 is not modified by NEDD8. WT and NEDP1 KO U2OS cells were mock transfected or transfected with FLAG-PARP-1. After low induction with doxycycline for 24 h, cells were harvested and immunoprecipitation was performed with anti-FLAG beads. After stringent washes, beads were split evenly and were treated with the deubiquitinase USP1/UAF1, the deneddylase GST-NEDP1 or left untreated. Bound proteins were then processed for Western blot analysis with the indicated antibodies. There is some modified PARP-1 that migrates as a higher molecular weight smear. The modification is reduced with deubiquitinase treatment but not with deneddylase treatment, which indicates that FLAG-PARP-1 is modified by ubiquitin. Furthermore, there is no immunoreactive of NEDD8 after immunoprecipitation, which indicates FLAG-PARP-1 is not modified by NEDD8 in either WT or NEDP1 KO cells.

C Reduction in PAR polymer accumulation in NEDP1 KO cells is not from increased PARG activity. U2OS WT and NEDP1 KO U2OS cells were pre-treated for 1 h with the cell permeable PARG inhibitor PDD 17273 (5 μM) before treatment with H_2O_2 (600 μM). Cells were treated for the indicated amount of time, lysed and prepared for Western blot analysis with α-PAR polymer antibody. PARG inhibition leads to increased PAR polymer accumulation in both cells lines as compared to cells without PARG inhibition (Fig 4A). However, the accumulation of PAR polymer in NEDP1 KO cells is still lower than in WT cells, which indicates PARP-1 is not fully activated in NEDP1 KO cells.

D NEDD8 is predominantly nuclear in both WT and NEDP1 KO cells. U2OS WT and NEDP1 KO cells were plated on glass coverslips, fixed and prepared for immunofluorescence detection with α-NEDD8 antibody and the DNA stain DAPI (scale bar = 10 μm).

E MNase digestion of DNA increases PARP-1-NEDD8 trimer binding. NEDP1 KO U2OS cells were harvested, and lysates were treated with DNase only or with DNase plus MNase. Treated lysates were then incubated with recombinant Zn1-GFP or Zn2-GFP for 1 h followed by immunoprecipitation with GFP-Trap. Immunoprecipitated proteins were resolved by SDS–PAGE followed by Western blot analysis with the indicated antibodies.

 © 2019 The Authors

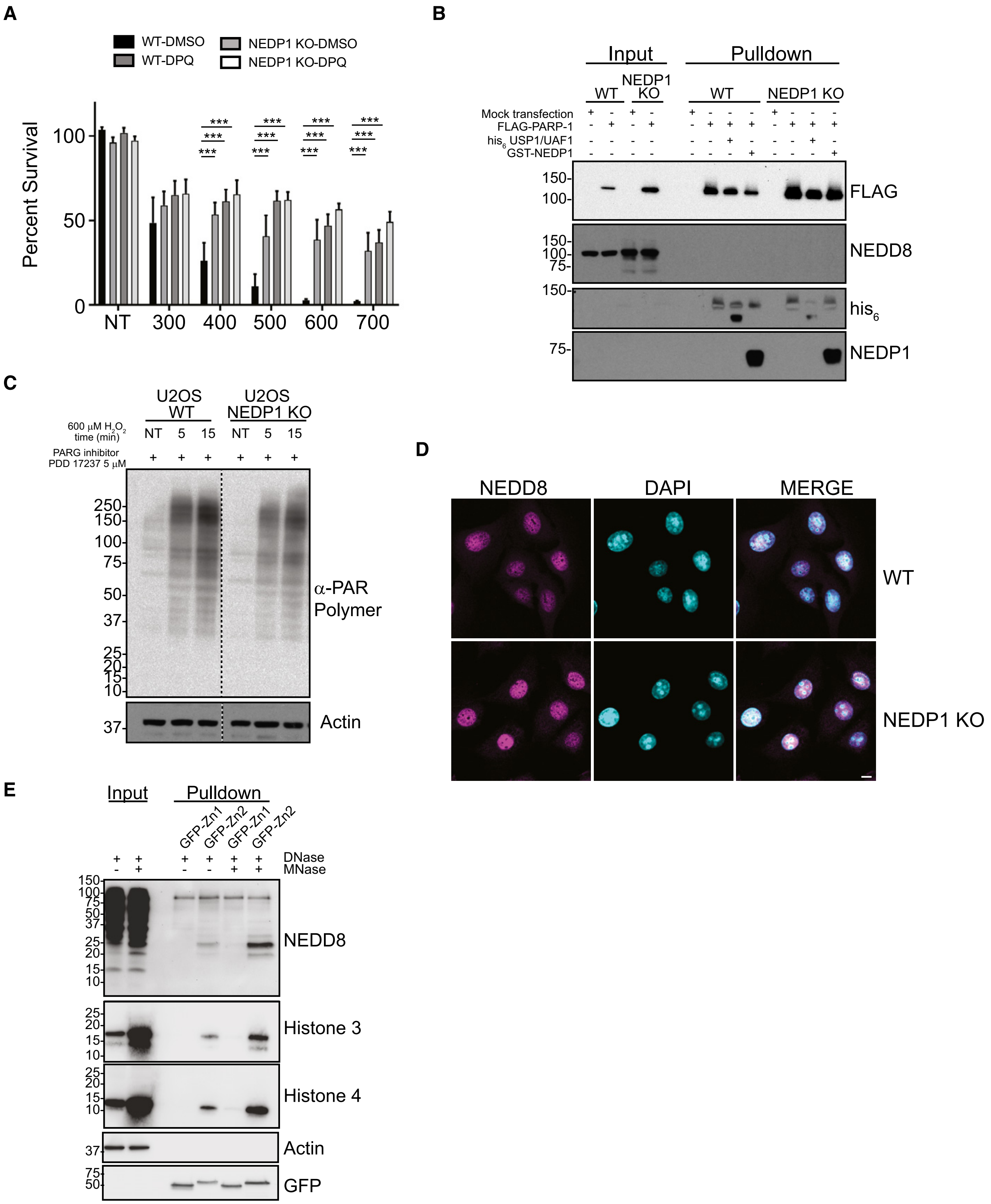
A
WT-DMSO
WT-DPQ
NEDP1 KO-DMSO
NEDP1 KO-DPQ
Percent Survival
100
50
0
***
NT
300
400
500
600
700
B
Input
Pulldown
WT
NEDP1 KO
Mock transfection
FLAG-PARP-1
his6 USP1/UAF1
GST-NEDP1
150-
100-
75-
FLAG
NEDD8
his6
NEDP1
C
U2OS WT
U2OS NEDP1 KO
600 μM H2O2 time (min)
NT 5 15 NT 5 15
PARG inhibitor PDD 17237 5 μM
250
150
100
75
50
37
25
20
15
10
α-PAR Polymer
Actin
D
NEDD8
DAPI
MERGE
WT
NEDP1 KO
E
Input
Pulldown
GFP-Zn1
GFP-Zn2
GFP-Zn1
GFP-Zn2
DNase
MNase
NEDD8
Histone 3
Histone 4
Actin
GFP

Figure EV4.

Figure EV5. NEDD8 trimers bind to the DNA-binding domain of PARP-1 to regulate PARP-1-dependent gene expression.

A Expression of TNF-α induced and PARP-1-dependent gene CXCL10 is increased in NEDP1 KO cells. WT, NEDP1 KO and CAND1 KO HEK 293 cells were plated in 12-well plates. Twenty-four hours later, cells were left untreated or treated with TNF-α (10 ng/ml) for 4 h, and then, RNA was harvested. cDNA was generated from the RNA, and then, qPCR was performed with primers for IκBα, CXCL10 and 18S. The TNF-α induced expression of IκBα or CXCL10 was compared to stable 18S transcription. Fold induction for NEDP1 KO and CAND1 KO cells was then normalized to the induction in WT for each experiment. Graphs represent mean ± SEM of the normalized induction from each replicate. ANOVA with Bonferroni *post hoc* test: $n = 3$, *$P < 0.033$, n.s. denotes not statistically significant.

B Increased expression of CXCL10 following TNF-α stimulation in NEDP1 KO cells is rescued by re-expression of WT NEDP1. WT or NEDP1 KO HEK 293 cells plated in 12-well plates and transfected with empty vector or p-CMV NEDP1. Forty-eight hours later, cells were left untreated or treated with TNF-α (10 ng/ml) for 4 h, and then, RNA was harvested. cDNA was generated from the RNA, and then, qPCR was performed with primers for CXCL10 and 18S. The TNF-α induced expression of CXCL10 compared to stable 18S transcription. Fold induction for NEDP1 KO was then normalized to the induction in empty vector transfected WT cells for each experiment. Graphs represent mean ± SEM of the normalized induction from each replicate. ANOVA with Bonferroni *post hoc* test: $n = 3$, **$P < 0.0021$, n.s. denotes not statistically significant.

C GST and GST-PARP-1 Zn1 + 2 were purified from BL21 cells and resolved by SDS–PAGE and visualized with Coomassie stain. GST-PARP-1 Zn1 + 2 but not GST can enrich NEDD8 trimers from NEDP1 KO lysate.

D 2D gel electrophoresis of GST pulldowns from NEDP1 KO lysate followed by Western blot analysis with α-NEDD8 antibody indicates that the NEDD8 trimers in Fig 6B are specific to GST-PARP-1 Zn1 + Zn2 pulldown only.

E Diagram which depicts the evolutionary conservation of lysines residues (blue text) in mature NEDD8 from *Caenorhabditis elegans* to *Homo sapiens*.

© 2019 The Authors

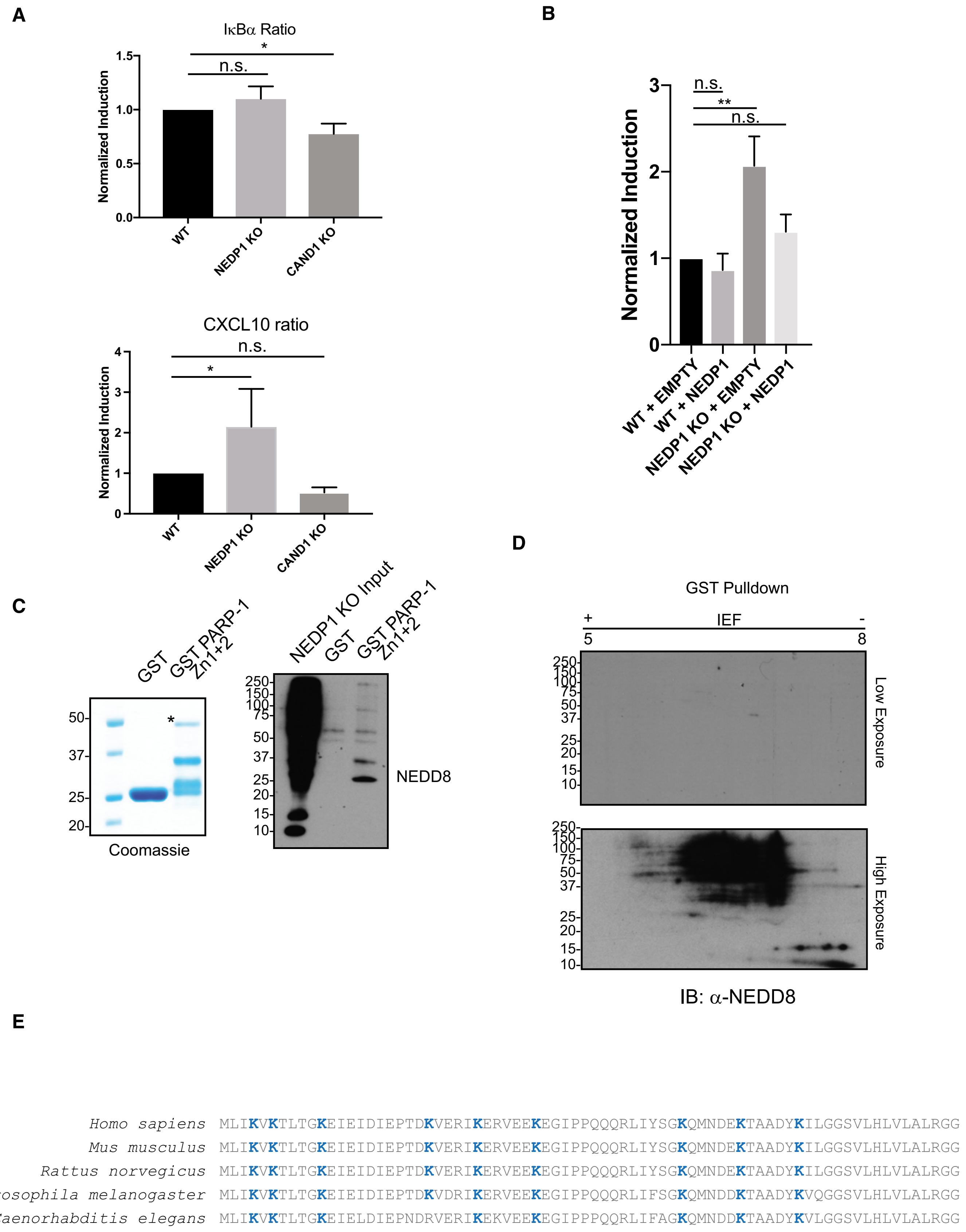
A
IκBα Ratio
Normalized Induction
n.s.
*
WT
NEDP1 KO
CAND1 KO
CXCL10 ratio
B
Normalized Induction
n.s.
**
WT + EMPTY
WT + NEDP1
NEDP1 KO + EMPTY
NEDP1 KO + NEDP1
C
GST
GST PARP-1 Zn1+2
Coomassie
NEDP1 KO Input
NEDD8
D
GST Pulldown
IEF
Low Exposure
High Exposure
IB: α-NEDD8
E
Homo sapiens MLIKVKTLTGKEIEIDIEPTDKVERIKERVEEKEGIPPQQQRLIYSGKQMNDEKTAADYKILGGSVLHLVLALRGG
Mus musculus MLIKVKTLTGKEIEIDIEPTDKVERIKERVEEKEGIPPQQQRLIYSGKQMNDEKTAADYKILGGSVLHLVLALRGG
Rattus norvegicus MLIKVKTLTGKEIEIDIEPTDKVERIKERVEEKEGIPPQQQRLIYSGKQMNDEKTAADYKILGGSVLHLVLALRGG
Drosophila melanogaster MLIKVKTLTGKEIEIDIEPTDKVDRIKERVEEKEGIPPQQQRLIFSGKQMNDDKTAADYKVQGGSVLHLVLALRGG
Caenorhabditis elegans MLIKVKTLTGKEIELDIEPNDRVERIKEKVEEKEGIPPPQQRLIFAGKQMNDDKTAADYKVLGGSVLHLVLALRGG

Figure EV5.
