# Supplementary material for: Unanchored tri‐NEDD8 inhibits PARP‐1 to protect from oxidative stress‐induced cell death
Source: EMBO J. 2019 Feb 25;38(6):e100024. doi: 10.15252/embj.2018100024 (PMC6418418; doi:10.15252/embj.2018100024)
Supplement: Supplementary file 2 — Table EV1 [file EMBJ-38-e100024-s002.docx]

| **Top proteins identified by label-free quantitative mass spectrometry.** | | | | |
| --- | --- | --- | --- | --- |
| **Rank** | **Protein names** | **Fold Enrichment CA/DAGC** | **HALO-NEDP1 CA log2(iBAQ)** | **ADP-Ribose**  **Substrate** |
| 1 | NEDD8 * | 50 | 30.9 |  |
| 2 | UBE2M * | 691 | 27.0 |  |
| 3 | Nucleophosmin | 16 | 25.9 | x |
| 4 | DCUN1D1 * | 156 | 25.0 |  |
| 5 | Small nuclear ribonucleoprotein Sm D1 | 15 | 24.9 | x |
| 6 | Nucleolin | 33 | 24.2 | x |
| 7 | PARP-1 | 200 | 24.0 | x |
| 8 | ATP-dependent 6-phosphofructokinase muscle type | 155 | 23.7 | x |
| 9 | Acyl-coenzyme A oxidase-like protein | 162 | 22.2 |  |
| 10 | Nucleolar and coiled-body phosphoprotein 1 | 60 | 22.2 | x |
| 11 | NSUN2 | 302 | 22.2 |  |
| 12 | UBE2N | 31 | 22.0 |  |
| 13 | UBA3 * | 20 | 21.9 |  |
| 14 | CSN8 * | ∞ | 21.8 |  |
| 15 | CAND1 * | 55 | 21.8 | x |
| 16 | ULA1 * | 83 | 21.7 |  |
| 17 | Heterogeneous nuclear ribonucleoprotein U | 26 | 21.5 | x |
| 18 | Nucleolar RNA helicase 2 | 177 | 21.3 | x |
| 19 | XRCC5 | 41 | 21.2 | x |
| 20 | Cullin-2 * | 19 | 21.1 |  |
| 21 | Mitochondrial import receptor subunit TOM20 homolog | ∞ | 21.1 | x |
| 22 | CSN6 * | 203 | 20.9 |  |
| 23 | Probable ATP-Dependent RNA Helicase DDX46 | 44 | 20.8 |  |
| 24 | Putative uncharacterized protein PNAS-138 | ∞ | 20.7 |  |
| 25 | CSN7A * | ∞ | 20.7 |  |
| 26 | CSN5 * | 167 | 20.6 |  |
| 27 | XRCC6 | 1318 | 20.6 | x |
| 28 | High mobility group protein B2 | 22 | 20.6 | x |
| 29 | DNA replication licensing factor MCM3 | 93 | 20.5 | x |
| 30 | General transcription factor II-I | 180 | 20.5 | x |
| 31 | DNA mismatch repair protein Msh2 | 67 | 20.5 | x |
| 32 | Replication factor C subunit 4 | 76 | 20.5 | x |
| 33 | U1 small nuclear ribonucleoprotein A | ∞ | 20.5 |  |
| 34 | DNA replication licensing factor MCM2 | 266 | 20.4 |  |
| 35 | Far upstream element-binding protein 2 | 18 | 20.4 | x |
| 36 | Splicing factor, proline- and glutamine-rich | 37 | 20.4 | x |
| 37 | U2 snRNP-associated SURP motif-containing protein | 63 | 20.4 |  |
| 38 | Lamina-associated polypeptide 2, isoform alpha | 56 | 20.4 | x |
| 39 | Replication factor C subunit 5 | ∞ | 20.2 | x |
| 40 | DNA replication licensing factor MCM4 | 100 | 20.1 | x |

**Table EV I**

**Table EV I Top proteins identified by mass spectrometry analysis.**

Table of the top 40 proteins identified by mass spectrometry analysis in Figure 2A. Proteins with a greater than 15-fold enrichment ratio based on the iBAQ intensities from the HALO-NEDP1 CA pulldown over the HALO-NEDP1 DAGC pulldown are ranked according to the iBAQ intensity identified from the HALO-NEDP1 CA pulldown. Proteins that are components of the NEDD8 pathway are denoted with an asterisk. Proteins identified as being modified by ADP-ribosylation (Vivelo *et al.*, 2017) are denoted with and x.
